# Rapid Aggravation of Rhabdomyolysis Caused by Daptomycin After Aortic Arch Replacement: A Case Report

**DOI:** 10.7759/cureus.53611

**Published:** 2024-02-05

**Authors:** Kenichi Takechi, Mayuko Fujimoto, Tomoko Abe, Ichiro Shimizu

**Affiliations:** 1 Department of Anesthesia, Matsuyama Red Cross Hospital, Matsuyama, JPN

**Keywords:** lower limb ischemia, creatine kinase, daptomycin, cardiopulmonary bypass, rhabdomyolysis

## Abstract

Although rare, rhabdomyolysis is a serious complication of cardiothoracic surgery. Daptomycin is a polypeptide antimicrobial agent used to treat methicillin-resistant *Staphylococcus aureus* (MRSA) infections of the soft tissues. Daptomycin is associated with elevations in serum creatine kinase (CK).

A 50-year-old man with acute Stanford A-type aortic dissection was performed Bentall procedure and total arch replacement with frozen elephant trunk. The CK level was 6,573 U/L on the first postoperative day (POD), suggesting rhabdomyolysis associated with lower limb ischemia. The CK level increased to 11,934 U/L on POD 2 and started to decrease thereafter. On POD 5, the patient had a suspected surgical site infection. Antibiotics were changed to empiric therapy of daptomycin and meropenem to address soft tissue MRSA infection. The CK level at the start of daptomycin administration was 4,122 U/L. However, the CK level rose to 21,813 U/L on POD 6. None of the findings suggested new-onset lower limb ischemia. Assuming that the rhabdomyolysis was induced by daptomycin, it was discontinued. The CK level peaked at 26,123 U/L on POD 8, after which it started to decrease and normalized on POD 16.

Daptomycin should be used with extreme caution in patients recovering from rhabdomyolysis.

## Introduction

Although rare, rhabdomyolysis is a serious complication of cardiothoracic surgery with cardiopulmonary bypass [1]. The drugs used during cardiovascular surgery can potentially cause rhabdomyolysis [2]. Daptomycin is a polypeptide antimicrobial agent used to treat methicillin-resistant *Staphylococcus aureus* (MRSA) infections of the skin and soft tissues; daptomycin is associated with elevations in serum creatine kinase (CK) [3].

We present a case of rhabdomyolysis after total aortic arch replacement that flared up and worsened after the administration of daptomycin during the rhabdomyolysis recovery period.

This manuscript adheres to the applicable EQUATOR (Enhancing the QUAlity and Transparency Of health Research) guidelines. The patient provided written informed consent for publishing this case report.

## Case presentation

A 50-year-old man (height, 172 cm; weight, 100 kg; body mass index, 33.8 kg/m^2^) with acute Stanford A-type aortic dissection was brought to the emergency department. Preoperative contrast-enhanced computed tomography revealed aortic dissection from the sinus of Valsalva to the iliac artery bifurcation, poor contrast in the left common iliac artery due to thromboembolic occlusion, and left renal infarction. Preoperative blood examination revealed renal injury, with a creatinine level of 1.67 mg/dL and a serum CK level of 170 U/L (Table 1).

**Table 1:** Laboratory findings at admission WBC: white blood cell; RBC: red blood cell; Hb: hemoglobin; Plt: platelet; AST: aspartate aminotransferase; ALT: alanine aminotransferase; LD: lactate dehydrogenase; BUN: blood urea nitrogen; Cre: creatinine; CK: creatine kinase

Test	Patient's Result	Reference Interval
WBC count	15980 /μL	3000-9600 /μL
RBC count	468×10^4^ /μL	400-552×10^4^ /μL
Hb	15.4 g/dL	13.2-17.2 g/dL
Plt count	16.6 ×10^4^ /μL	14.8-36.1×10^4^ /μL
AST	40 U/L	13-33 U/L
ALT	33 U/L	8-42 U/L
LD	405 U/L	119-229 U/L
BUN	20.1 mg/dL	8-20 mg/dL
Cre	1.67 mg/dL	0.61-1.04 mg/dL
CK	170 U/L	62-287 U/L

Bentall procedure and total arch replacement with frozen elephant trunk were planned. Anesthesia was induced using midazolam (5 mg), fentanyl (0.5 mg), and rocuronium (100 mg) and maintained using target control infusion of propofol (2-3 μg/mL), remifentanil (0.1-0.2 μg·kg^-1·^min^-1^), and rocuronium (5 μg·kg^-1^·min^-1^). Cefazolin (1 g) was administered prior to skin incision, followed by additional doses every 8 hours. After anesthesia induction and starting surgery, arterial and venous cannulas were inserted through the right femoral artery and right atrium respectively. Furthermore, cooling was initiated immediately after instituting cardiopulmonary bypass. Subsequently, deep hypothermia was induced, the aorta was incised after circulatory arrest, and selective antegrade cerebral perfusion was established. After a frozen elephant trunk was inserted, body circulation was resumed.

**Figure 1:**
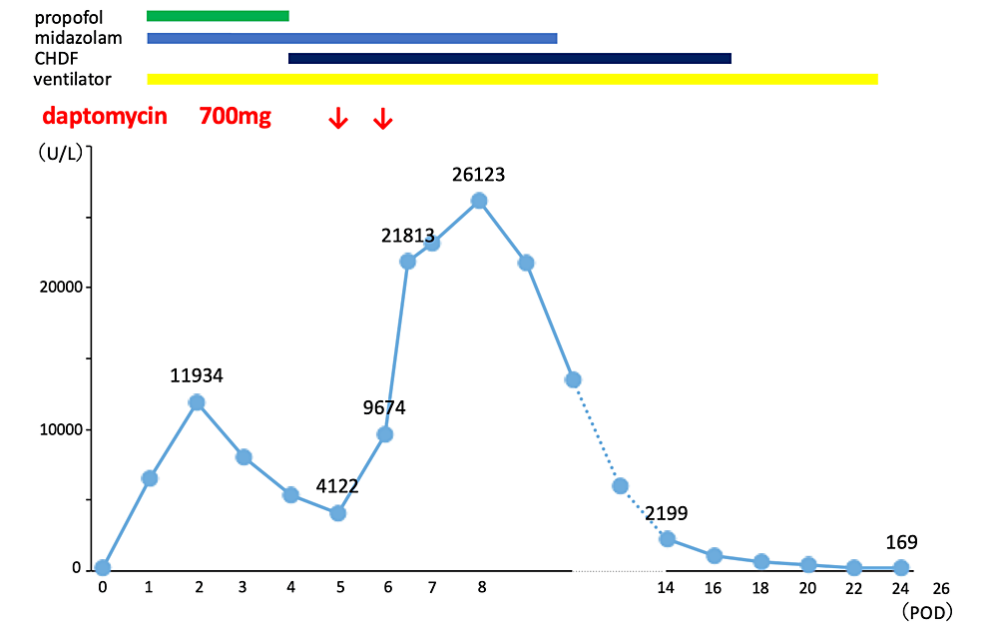
Trends in serum creatine kinase levels during intensive care unit stay CHDF, continuous hemodiafiltration; POD, postoperative day

The total operation, cardiopulmonary bypass, and selective antegrade cerebral perfusion times were 1019 minutes, 458 minutes, and 120 minutes, respectively. Postoperatively, the patient was transferred to the intensive care unit (ICU), where he was kept on mechanical ventilation. The CK level was 6,573 U/L on the first postoperative day (POD), suggesting rhabdomyolysis associated with lower limb ischemia. The CK level increased to 11,934 U/L on POD 2 and started to decrease thereafter. Continuous hemodiafiltration (CHDF) was initiated on POD 4, owing to worsening renal function and decreased urine output. Since the patient continually had a fever in the postoperative period and the white blood cell count flared up (16,230/μL to 23,670/μL) on POD 4 to POD 5. Sputum and blood cultures were negative, suggesting a possible surgical site infection, including mediastinitis. Antibiotics were changed to empiric therapy of daptomycin and meropenem to address soft tissue MRSA infection. Daptomycin was administered at a dose of 7 mg/kg, and the CK level at the start of daptomycin administration was 4,122 U/L. However, the CK level rose to 9,674 U/L on POD 6, increasing to 21,813 U/L on the same day (Figure 1).

None of the findings suggested new-onset lower limb ischemia or compartment syndrome. No drugs, such as haloperidol, were administered that could cause the malignant syndrome. Propofol was discontinued on POD 4. Assuming that the rhabdomyolysis was induced by daptomycin, it was discontinued. The CK level peaked at 26,123 U/L on POD 8, after which it started to decrease and normalized on POD 16. The patient was weaned off the CHDF and ventilator on POD 17 and POD 21, respectively, and discharged from the ICU on POD 26. The patient experienced palsy of the lower limbs, muscle weakness throughout the body, and dysphagia, which gradually improved. He was transferred to the hospital for rehabilitation approximately 3 months postoperatively.

## Discussion

Numerous factors may raise the propensity of rhabdomyolysis after cardiac surgery. Direct femoral artery cannulation, arterial diseases, long extracorporeal circulation, low cardiac output syndrome, and continuous epinephrine infusion have been described as risk factors of rhabdomyolysis [1]. Ischemic myopathy of the lower extremities causes severe complications, such as metabolic acidosis, myoglobinuria, hypercalcemia, and acute renal failure. In this case, preoperative occlusion of the left femoral artery due to aortic dissection and cannulation of the right femoral artery for cardiopulmonary bypass may have contributed to the ischemia and rhabdomyolysis in the lower extremities. Additionally, the circulatory arrest with deep hypothermia may have contributed. Although the safety limits of deep hypothermic circulatory arrest have been discussed, they were mainly regarding neurological complications; its safety limits and effects on the skeletal muscles remain controversial [4]. Positioning injuries are more frequent in obese cardiac surgical patients because obese patients are at a high risk of pressure injuries, such as rhabdomyolysis and compartment syndrome [5]. In this case, lower limb ischemia, hypothermic circulatory arrest, and obesity may have contributed to the development of postoperative rhabdomyolysis.

In this patient, rhabdomyolysis improved once and then flared up. Drug-induced rhabdomyolysis has been reported with various drugs; however, daptomycin was the most likely cause of the rhabdomyolysis relapse in this case. A typical adverse reaction of daptomycin is CK elevation, which was reported in 6.7% of patients in a Phase 3 study [6]. In previous reports, CK elevation due to daptomycin was observed 10 days after its administration [7]. There have been no reports of rhabdomyolysis occurring immediately after daptomycin administration, as observed in this case.

Daptomycin undergoes renal excretion, and patients with renal dysfunction may have an increased risk of developing adverse effects due to increased daptomycin levels in the blood. A correlation between the lowest daptomycin concentration and elevated CK levels has been reported [8]. This patient showed renal impairment during daptomycin initiation. However, CHDF was introduced at the start of daptomycin treatment, as it reportedly decreases the concentration of daptomycin in the blood [9]. Therefore, it was unlikely that the high concentration of daptomycin could have flared up the rhabdomyolysis.

The mechanism underlying the detrimental effects of daptomycin on skeletal muscles has not been elucidated. In bacteria, daptomycin enters the cytoplasmic membrane via calcium-dependent mechanisms, causing membrane dysfunction and cell death. A proposed mechanism for daptomycin-induced rhabdomyolysis is that daptomycin damages the cell membrane through the gap in the outer layer of the sarcolemma. This causes the calcium to leak from the extracellular compartment into the muscle fibers, activating a downstream mechanism that leads to cell death [10]. In this case, the cell membrane of the skeletal muscle was damaged due to lower limb ischemia and obesity-induced contusion. Moreover, the skeletal muscle was in the process of repair. Therefore, this may have increased the susceptibility of skeletal muscle to daptomycin, resulting in the rapid recurrence of rhabdomyolysis after daptomycin administration.

## Conclusions

We experienced a case of rapid aggravation of rhabdomyolysis after the use of daptomycin in a patient with rhabdomyolysis after cardiac surgery. Daptomycin should be used with extreme caution in patients recovering from rhabdomyolysis. When daptomycin is administered in such patients, the CK levels should be measured over time to monitor rhabdomyolysis recurrence. In addition, if rhabdomyolysis flares up, daptomycin should be discontinued immediately.

## References

[1] Omar AS, Ewila H, Aboulnaga S, Tuli AK, Singh R (2016). Rhabdomyolysis following cardiac surgery: a prospective, descriptive, single-center study. Biomed Res Int.

[2] Zimmerman JL, Shen MC (2013). Rhabdomyolysis. Chest.

[3] Teng C, Baus C, Wilson JP, Frei CR (2019). Rhabdomyolysis associations with antibiotics: a pharmacovigilance study of the FDA Adverse Event Reporting System (FAERS). Int J Med Sci.

[4] Tanaka A, Chehadi M, Smith HN (2023). Deep hypothermic circulatory arrest with retrograde cerebral perfusion: how long is safe?. Ann Thorac Surg.

[5] Chacon MM, Cheruku SR, Neuburger PJ, Lester L, Shillcutt SK (2018). Perioperative care of the obese cardiac surgical patient. J Cardiothorac Vasc Anesth.

[6] Samura M, Hirose N, Kurata T (2021). Identification of risk factors for daptomycin-associated creatine phosphokinase elevation and development of a risk prediction model for incidence probability. Open Forum Infect Dis.

[7] Byren I, Rege S, Campanaro E, Yankelev S, Anastasiou D, Kuropatkin G, Evans R (2012). Randomized controlled trial of the safety and efficacy of Daptomycin versus standard-of-care therapy for management of patients with osteomyelitis associated with prosthetic devices undergoing two-stage revision arthroplasty. Antimicrob Agents Chemother.

[8] Bhavnani SM, Rubino CM, Ambrose PG, Drusano GL (2010). Daptomycin exposure and the probability of elevations in the creatine phosphokinase level: data from a randomized trial of patients with bacteremia and endocarditis. Clin Infect Dis.

[9] Preiswerk B, Rudiger A, Fehr J, Corti N (2013). Experience with daptomycin daily dosing in ICU patients undergoing continuous renal replacement therapy. Infection.

[10] Kostrominova TY, Hassett CA, Rader EP (2010). Characterization of skeletal muscle effects associated with daptomycin in rats. Muscle Nerve.

